# Cri-Du-Chat Syndrome Associated With Meningomyelocele: A Case Report

**DOI:** 10.7759/cureus.46279

**Published:** 2023-09-30

**Authors:** Fatimah A Alabbad, Roqaia Alali, Mohammed Alquraini, Zahra M Alghannam, Mohammed B Alabdullah, Haider H AlMousa

**Affiliations:** 1 Neonatology Department, Maternity and Children Hospital, Alahsa, SAU; 2 Paediatrics and Child Health Department, King Faisal University, Hofuf, SAU; 3 Internal Medicine Department, Alahsa Health Cluster, Alahsa, SAU; 4 Pediatrics Department, Maternity and Children Hospital, Alahsa, SAU

**Keywords:** spina bifida, neonatal, meningomyelocele, cri-du-chat, case report

## Abstract

Cri-du-chat syndrome (CdCS) is a rare genetic disorder in which the short arm of chromosome 5 is deleted. This report aims to highlight a rare association with the syndrome. We present a preterm male delivered at 35 weeks gestation with an antenatal diagnosis of meningomyelocele. The patient's clinical examination revealed ruptured lumbosacral meningomyelocele, lower limb hypotonia, and hyporeflexia. The patient also displayed dysmorphic features, including microcephaly, a rounded face, low-set ears, and club feet. In addition, he is noted to have a high-pitched cry. Diagnosis of Chiari tonsil hernia type II was made by magnetic resonance imaging, and whole exome sequencing has confirmed CdCS. The spina bifida was surgically corrected, and the patient has since been cared for by a multidisciplinary team. The patient's short-term follow-up revealed a significant developmental delay. Few cases of CdCS associated with meningomyelocele have been reported. More evidence is needed to support a relevant association between CdCS and meningomyelocele.

## Introduction

Cri-du-chat syndrome (CdCS) is also known as 5p syndrome and cat-like crying syndrome (OMIM#123450). It is a rare genetic disorder caused by a deletion of genetic material on the short arm of chromosome 5. The incidence of CdCS ranges from approximately one in 15,000-50,000 live births [1], but later authors changed this estimate to 1:37,000 live births [2].

The hallmark features of CdCS include a distinctive high-pitched monotonous cat-like crying, along with low birth weight, microcephaly, hypotonia, impaired growth, and developmental delay. Specific craniofacial features include a rounded face, bilateral epicanthal folds, hypertelorism, a broad nasal bridge, down-slanting palpebral fissures, and a short philtrum. CdCS is also linked to variable intellectual disabilities and neuropsychiatric manifestations, including autistic behaviors. In some cases, individuals with CdCS may also exhibit retinal vessel abnormalities, optic atrophy, or congenital renal and heart defects. Furthermore, a correlation has been observed between CdCS syndrome and central nervous malformation. Nevertheless, instances of neural tube defects (NTDs) are infrequently detected within this syndrome [3-4].

From a diagnostic perspective, a karyotype analysis is among the initial diagnostic tests used to confirm the condition. However, when there is strong clinical suspicion, despite a normal karyotype result, additional targeted tests, such as fluorescence in situ hybridization (FISH), comparative genomic hybridization (CGH), or quantitative polymerase chain reaction (PCR), can be conducted [5].

Regarding management, as there is currently no specialized treatment available for CdCS, active involvement in early rehabilitative and educational interventions has demonstrated the potential to improve the long-term prognosis. It is worth highlighting that substantial progress has been made in supporting the social adjustment of individuals living with this condition [2].

NTDs refer to congenital abnormalities of the central nervous system that occur when the neural tube does not properly close during the early stages of embryo formation. Among these, spina bifida and anencephaly are the most prevalent and debilitating types [6]. The occurrence of NTDs varies, with prevalence rates spanning from 0.5 to over 10 cases per 1,000 pregnancies, indicating a significant global presence among birth abnormalities. Syndromic cases account for fewer than 10% of all NTD instances [7-8].

To the best of our knowledge, this case represents one of the few reported cases of CdCS associated with lumbosacral meningomyelocele, and, notably, it is the initial documented occurrence from Saudi Arabia.

## Case presentation

The patient was a preterm male and the first product of a non-consanguineous marriage to a 20-year-old prim gravida lady and a 23-year-old father, both of whom had insignificant medical histories. The family history did not include any cases of syndromes or hereditary disorders. Throughout her pregnancy, the mother consistently attended her prenatal appointments, adhered to her prescribed supplements, and had no known exposure to teratogenic substances. Antenatal follow-up was significant for spina bifida, which was diagnosed at gestation of 33 weeks. The patient was born at gestation of 35 weeks, via cesarean section, after prolonged premature rupture of membranes and fetal distress. Following birth, he did not require more than initial steps, given an Apgar score of 6 and 8 at the first and fifth minutes, respectively. The patient weighed 2,040 g, which falls at the 14th percentile for his gestational age. He measured 48 cm in length, corresponding to the 79th percentile, while his head circumference was 28 cm, below the 10th percentile for his gestational age [9].

The clinical assessment revealed an alert and responsive newborn who displayed distinctive facial characteristics, including microcephaly, micrognathia, hypertelorism, a rounded face, low-set ears, an elongated philtrum, and a broad nasal bridge. The baby exhibited spontaneous and equal movement of all limbs, mild hypotonia, and reduced deep tendon reflexes in all four limbs. The Moro reflex was normal, while the sucking reflex was weak. Anal wrinkles were present, and sensory function appeared intact. On spine examination, a 4 x 4 cm ruptured membranous lumbosacral lump was found, discharging clear fluids and surrounded by non-erythematous intact skin. Furthermore, the musculoskeletal evaluation indicated bilateral talipes equinovarus. Examinations of the respiratory, cardiovascular, abdominal, and genitourinary systems did not reveal any notable findings.

**Figure 1:**
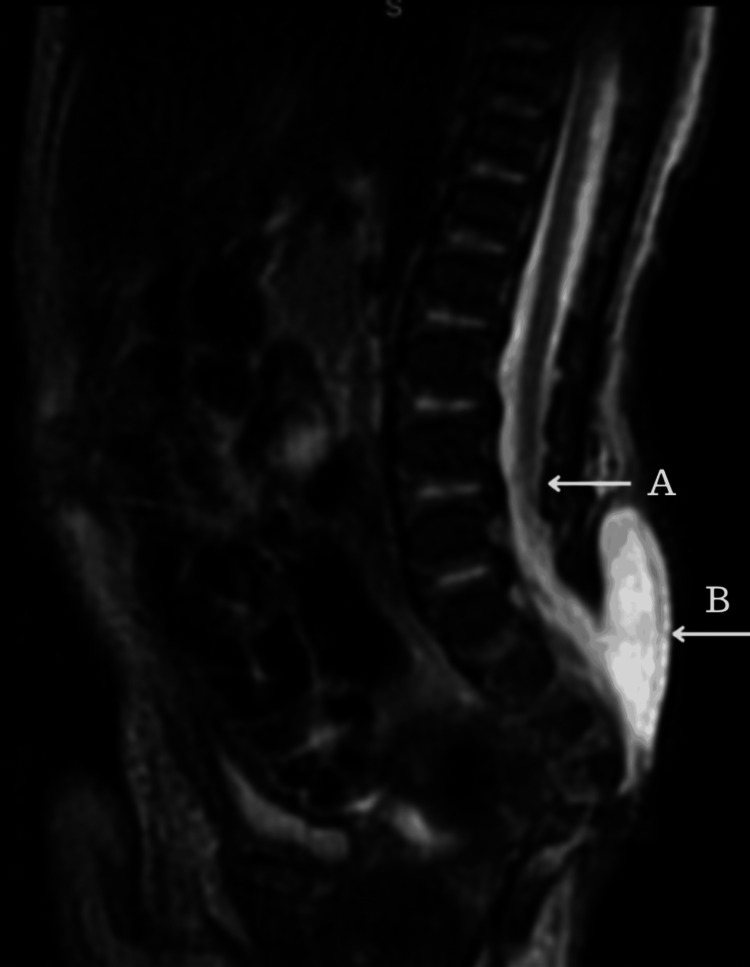
Lumbosacral spine MRI sagittal T2-weighted image demonstrates a tethered spinal cord (Arrow A) down to the meningomyelocele that is herniated through a large sacral bony defect (Arrow B).

**Figure 2:**
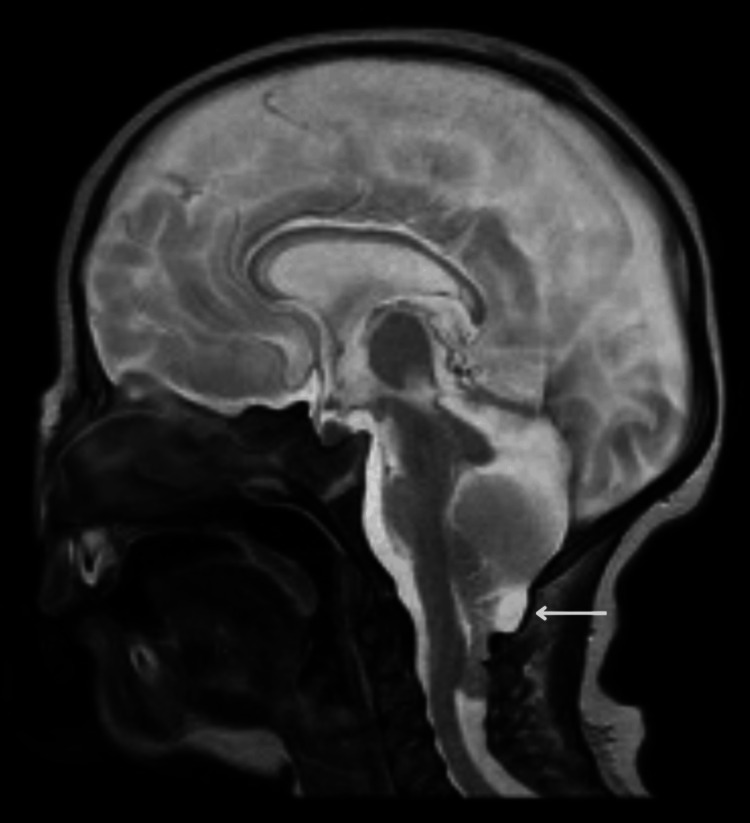
MRI brain midline sagittal T2-weighted MRI brain image that demonstrates a small posterior fossa with evidence of cerebellar tonsillar herniation through the crowded foramen magnum with a collapsed fourth ventricle.

**Figure 3:**
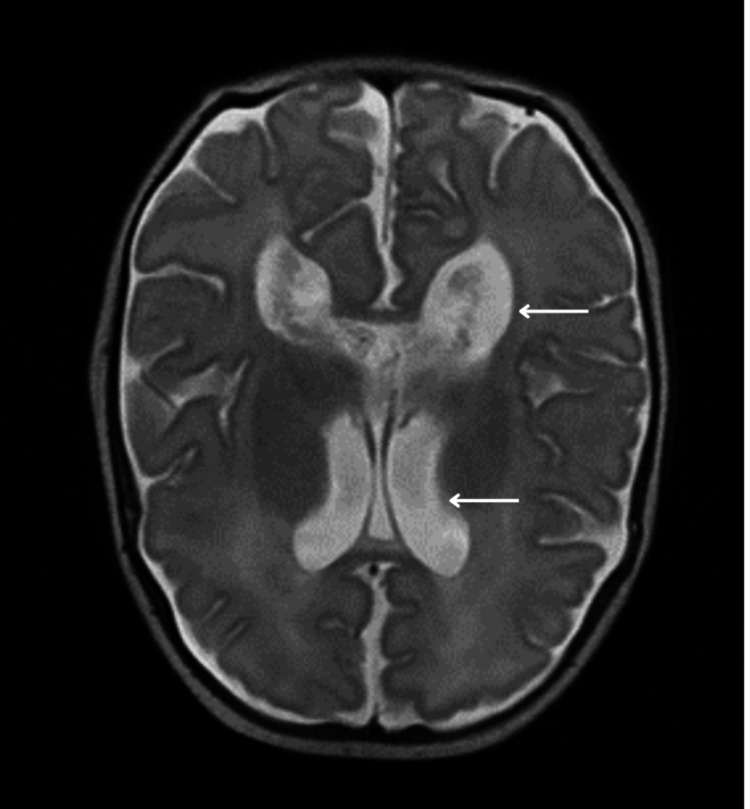
MRI brain axial T2-weighted MRI brain image that demonstrates mild dilatation of the lateral ventricles.

While in the neonatal intensive care unit (NICU), the initial laboratory tests yielded results within the expected range for the baby's gestational age. Subsequent brain and spinal magnetic resonance imaging confirmed the existence of lumbosacral meningomyelocele, along with a tethered cord (Figure 1). Additionally, Arnold-Chiari type II malformation and mild dilation of lateral ventricles were identified (Figures 2-3). As part of a comprehensive screening for additional congenital anomalies, ultrasound scans were conducted for the head, kidneys, and abdomen. These screenings identified slight enlargement of the lateral ventricles in the brain, but the remaining results were normal. Echocardiography also showed normal results.

The patient received broad-spectrum antibiotics and underwent surgical repair on the second day of life. Following the procedure, the patient's recovery in the hospital proceeded without complications. Throughout the hospitalization, the medical team observed that the infant consistently exhibited high-pitched crying. Given this distinctive crying pattern along with the previously mentioned clinical observations, suspicion arose regarding CdCS. Whole exome sequencing confirmed a loss of 33,742kb within the 5p15.33p13.2 chromosomal regions, establishing a diagnosis of CdCS.

## Discussion

This article explores an exceptionally rare combination of spina bifida alongside CdCS. The syndrome was first described in the medical literature in 1963 by Doctor Lejeune who named the disorder after the distinctive cat-like cry. It established that CdCS results from deletions of heterogeneous size in the short arm of chromosome 5. In over 80% of cases, this deletion is determined to be de novo, although it has been observed to be lower than previously reported, standing at 58.3% [10,11]. The majority of mutations arise from the paternal side attributed to the potential breakage of chromosome 5 during the formation of male gametes [10]. Approximately 15% of 5p deletions arise due to parental translocation between chromosome 5 and other chromosomes. Less common mechanisms, including mosaicism, inversions, or the presence of ring chromosomes, have also been suggested. For families with a translocation involving 5p deletion, the risk of having a child with CdCS ranged from 8.7% to 18.8% [12,13].

The primary clinical features evident at birth encompass a distinct plaintive and high-pitched monochromatic cry, reminiscent of a cat's mewing in 95.9% of cases [14]. Additional features include characteristic dysmorphic facial features [11-12,15-16], marked psychomotor and intellectual disability, and prenatal and postnatal growth delays [11]. The clinical spectrum and severity of the disease are influenced by the size of the deleted chromosomal region [14]. Our patient exhibits cardinal features of CdCS, including distinctive high-pitched crying, microcephaly, micrognathia, moon-shaped face, hypertelorism, broad nasal bridge, low-set ears, intrauterine growth restriction, and global neurodevelopmental delay.

In terms of neuroimaging, the most typical findings are brainstem hypoplasia and cerebellar white matter atrophy. Other reported brain anomalies consist of thinning of the corpus callosum, Dandy-Walker malformation, middle cerebellar peduncles hypoplasia, and dilated lateral and fourth ventricles [2,17]. In our case, alongside the relatively common findings of dilated lateral ventricles, he also manifested small posterior fossa, Arnold-Chiari II malformation, and meningomyelocele.

Schinzel reported a three-week-old girl with a large lumbosacral meningocele and chromosome 5 deletion [18]. Similarly, Mita et al. reported a 48-day-old girl with deletion of 5p, CdCS characteristics, and lumbosacral meningomyelocele [19]. In addition, Begleiter et al. described a newborn female with dysmorphic features, characterized by cat-like crying, who was diagnosed with both 5p deletion and meningomyelocele [20]. There have also been reports of recurrent anencephaly observed in sibling fetuses with partial monosomy 5p combined with other chromosomal aneuploidies. However, it has not been definitively determined whether these findings are linked to the combined chromosomal abnormalities or solely to the 5p deletion [18,21,22].

When delving into genetics, loss of genes such as methionine synthase reductase (MTRR) and mitochondrial ribosomal protein L36 (MRPL36) may potentially contribute to increased apoptosis and the occurrence of neural tube defects (NTDs) [23]. Moreover, a terminal deletion involving the short arm of chromosome 5 could encompass cadherin genes (CDH6, CDH9, CDH10, CDH12, CDH18) and Iroquois homeobox genes (IRX1, IRX2, IRX4). These genes have essential roles in processes like neurogenesis, neuron migration, and axon growth [24-25]. In our patient's genetic results, a deletion of 33,742 kb was identified within the chromosomal regions 5p15.33p13.2, which includes the 5p15 terminal region, aligning with findings seen in most patients. Interestingly, our patient deletion was found to include all the aforementioned genes (cadherin, IRX, MTRR, and MRPL 36), which could potentially be associated with the specific observation of NTDs [23,26].

## Conclusions

In summary, the occurrence of neural tube defects in individuals with CdCS is exceptionally rare and could potentially be overlooked or not adequately documented. Currently, there is insufficient data to establish a definitive pertinent link between neural tube defects and CdCS.

Future investigations involving molecular analysis of a larger cohort of CdCS patients presenting with neural tube defects are necessary to elucidate the specific genes responsible for this association. This, in turn, could carry important implications for both diagnosis and prognosis.
